# Evolutionary trends of respiratory syncytial viruses: Insights from large-scale surveillance and molecular dynamics of G glycoprotein

**DOI:** 10.1016/j.heliyon.2024.e30886

**Published:** 2024-05-09

**Authors:** Muhammad Nabeel Amjad, Jing Wang, Muhammad Awais Ashraf, Bei Shen, Ghayyas ud Din, Muhammad Asif Raza, Muhammad Shoaib, Lihuan Yue, Lingdie Chen, Huiting Xu, Wei Dong, Yihong Hu

**Affiliations:** a CAS Key Laboratory of Molecular Virology & Immunology, Institutional Center for Shared Technologies and Facilities, Pathogen Discovery and Big Data Platform, Shanghai Institute of Immunity and Infection, Chinese Academy of Sciences, Yueyang Road 320, Shanghai, 200031, China; b University of Chinese Academy of Sciences, Beijing, China; c Key Laboratory of New Animal Drug Project, Gansu Province/Key Laboratory of Veterinary Pharmaceutical Development, Ministry of Agriculture and Rural Affairs, Lanzhou Institute of Husbandry and Pharmaceutical Sciences of CAAS, Lanzhou, 730050, China; d Pediatric Department, Nanxiang Branch of Ruijin Hospital, Shanghai, 201802, China

**Keywords:** Phylogenetics, Molecular evolution, RNA virus, Respiratory syncytial virus, Central conserved region, Cysteine-mediated cross-linking

## Abstract

Human respiratory syncytial virus (RSV) is an underlying cause of lower respiratory illnesses in children, elderly and immunocompromised adults. RSV contains multiple structural and non-structural proteins with two major glycoproteins that control the initial phase of infection, fusion glycoprotein and the attachment (G) glycoprotein. G protein attaches to the ciliated cells of airways initiating the infection. The hypervariable G protein plays a vital role in evolution of RSV strains. We employed multiple bioinformatics tools on systematically accessed large-scale data to evaluate mutations, evolutionary history, and phylodynamics of RSV. Mutational analysis of central conserved region (CCR) on G protein-coding sequences between 163 and 189 positions revealed frequent mutations at site 178 in human RSV (hRSV) A while arginine to glutamine substitutions at site 180 positions in hRSV B, remained prevalent from 2009 to 2014. Phylogenetic analysis indicates multiple signature mutations within G protein responsible for diversification of clades. The USA and China have highest number of surveillance records, followed by Kenya. Markov Chain Monte Carlo Bayesian skyline plot revealed that RSV A evolved steadily from 1990 to 2000, and rapidly between 2003 and 2005. Evolution of RSV B continued from 2003 to 2022, with a high evolution stage from 2016 to 2020. Throughout evolution, cysteine residues maintained their strict conserved states while CCR has an entropy value of 0.0039(±0.0005). This study concludes the notion that RSV G glycoprotein is continuously evolving while the CCR region of G protein maintains its conserved state providing an opportunity for CCR-specific monoclonal antibodys (mAbs) and inhibitors as potential candidates for immunoprophylaxis.

## Highlights

• The conserved region in G protein, 163 and 189 amino acids has an entropy of 0.0039.
• RSV effective population size increased abruptly after 2010 on Bayesian skyline plot.
• 2009–2014, mutations at 178th in RSV A and 180th position in RSV B were prevalent.
• RSV has been recorded most frequently in China and the United States.

## 1 Introduction

Respiratory syncytial virus (RSV) was discovered in 1955 in a chimpanzee. Hailing from a family of *Paramyxoviridae*, RSV shares close genetic resemblance with other pneumonia viruses having single-stranded, negative sense linear RNA genome. Human respiratory syncytial virus (hRSV) contains two serotypes i.e., A and B, due to extensive mutations in their genome, particularly in glycoprotein G, which is an active constituent in viral infection and propagation [1]. HRSV consists of several structural and non-structural proteins that make the viral genome including small hydrophobic region, fusion glycoprotein, G glycoprotein, L protein, N nucleocapsid, matrix protein M, non-structural (NS1, NS2) proteins and phosphoprotein P. RSV G glycoprotein inclusively affects the disease progression by hindering host immune reflexes and suppressing the activity of mAbs against it [2]. G protein plays a crucial role in cell surface binding through CX3CR1 or GAGs, along with host response modulation [3]. Binding G protein with antibody hinders the disease progression and lower viral titers from the lungs were observed [4]. Further, G glycoprotein is also used for genotyping and strain classification in RSV. Those provide concrete bases for its application as a potential vaccine candidate along with fusion protein. RSV genotypes have been defined among the subgroup strains, GB1-4, BA1-10, URU1-2, and GB5-7 in RSV B and 13 genotypes for the subgroup A strains as SAA1, GA1-7, ON1-2, and NA1-4. The conditions used to define a genotype alter based on the phylogenetic studies inferred using multiple methods such as maximum parsimony, maximum likelihood, Bayesian inferences, or neighbor-joining [5].

RSV infection is considered a typical "childhood disease". It can also cause pneumonia, acute bronchiolitis, and other acute respiratory tract infections. RSV is a relative of influenza, measles, and the common cold viruses. Commonly causing acute lower respiratory infection in infants and young children, leading to pneumonia, acute bronchiolitis (inflammation of the small airways in the lungs), and even death in some cases [6]. Symptoms may include runny nose, nasal congestion, sneezing, watery eyes, dry cough, fever, and a mild sore throat, similar to those caused by other respiratory viruses, such as the common cold. The incubation period for RSV is usually one to three days but may range from about 12 to 36 hours. In most less severe cases, the patients recover in 5–7 days [7]. Treatment for RSV generally focuses on relieving symptoms through supportive care while the immune cells attack and neutralize the viral particles responsible for disease progression. However, severe cases may require hospitalization and treatment with oxygen or intravenous fluids that are part of supportive care treatment. Certain anti-viral therapies are also administered against RSV infections. In premature babies or those with underlying health conditions that weaken the immune system, RSV can be particularly fatal [8]. Ribavirin is a clinically approved drug for infants while Presatovir is in experimental trials [9].

Certain studies have claimed drug resistance towards both drugs because of extensive mutations in the RSV genome and a higher number of RSV strains [10]. In 2015, 33.1 million cases of RSV-associated acute lower respiratory infection were reported, 3.2 million people were admitted to the hospitals for RSV acute lower respiratory infection, and 59,600 deaths in children younger than five years were documented in hospitals with RSV-associated acute lower respiratory infection. Overall, 118, 200 deaths were recorded in hospitals and counted as high mortality rate estimates due to unavailability of the death data in the community [11]. Since 2017, new data on RSV cases in children have become readily available, including from many RSV surveillance studies to inspect RSV deaths in the community (RSV community mortality surveillance studies and child health and mortality surveillance CHAMPS [12] supported by the 10.13039/100000865Bill and Melinda Gates Foundation). Therefore, people who are at high risk, including infants and immunocompromised patients, need to take precautions against exposure to RSV, such as washing their hands frequently and avoiding contact with the sick.

This study explains the flow of mutations observed in the recorded sequences obtained from NCBI and GISAID till December 31, 2022. It provides a comprehensive approach for researchers to understand the genetic flow of the virus by unraveling the mutations responsible for new genotypes, conservancy of the central conserved region, and phylodynamics and geospatial distribution of the human respiratory syncytial virus.

## 2 Materials and methods

### 2.1 Sequence retrieval

All hRSV sequences available at NCBI and GISAID were downloaded along with their collection dates. We used hRSV A2 long strain (Accession No. M74568.1) and hRSV B 9320 (Accession No. AY353550.1) as reference strains [13]. G protein coding sequence (CDS) was extracted using MAFFT specific region extraction server (https://mafft.cbrc.jp/alignment/server/specificregion-last.html) based on LAST algorithm [14]. Phylogenetic and mutational analyses of hRSV were carried out using the final dataset of 2487 and 2079 non-identical sequences for hRSV A and hRSV B, respectively.

### 2.2 Phylogenetic analysis

Multiple sequence alignment was performed using the MAFFT online server with a cutoff value of 0.9. Fasttree –nt –gtr, Generalized Time Reversible plus Jukes-Cantor model was used to generate phylogenetic trees. We used TreeAnnotator v1.10.4 to annotate the phylogenetic tree with 10 % burn-in values. We used FigTree v1.4.4 for tree visualizations to highlight the critical genotypes.

### 2.3 Mutational analysis and entropy of CCR

We performed mutational analysis and entropy calculations for the central conserved region using BioEdit 7.2.5 version. Weblogos for the CCR region of hRSV A & B were generated using the Weblogo website (www.weblogo.berkeley.edu)

### 2.4 Bayesian skyline plot

We used BEAST 2.7.5 to conduct Bayesian skyline plot (BSP) analysis on the respiratory syncytial virus sequences. XML files were generated using Beauti2 with the best-fit GTR model having lognormal relaxed molecular clock distribution. The best-fit model was accessed from the datamonkey server (http://datamonkey.org/). Samples were recorded after every 1000 cycles in 50 million generations run. BSP was visualized using Tracer 1.7.2 with a bin size of 100.

### 2.5 Geographical distribution

Geographical data was extracted from the genome dataset using a Python snippet. ArcGIS® Pro v 3.1 was used to generate geographical distribution maps. A manually adjusted frequency-based color pallet was used to visualize the difference in frequencies.

### 2.6 Statistical analysis

Statistical analysis included Tajima's test of neutrality [15], which compares the number of segregating sites per site with the nucleotide diversity and p-distance calculations [16]. Both tests were performed using MEGA 11 to analyze the statistical significance of datasets [17].

## 3 Results

### 3.1 HRSV G conserved region

**Fig. 1:**
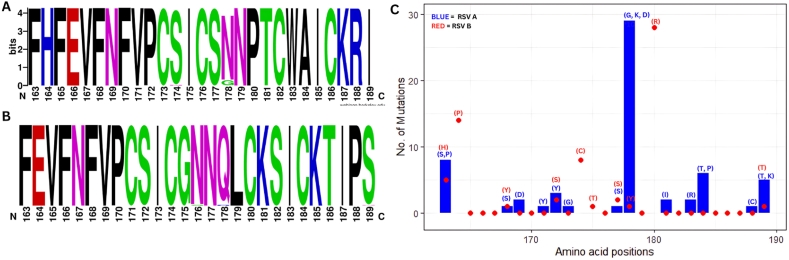
Weblogos of CCR **Note:** Weblogos of RSV A (A) and RSV B (B) ranging from 163 to 189 amino acid position while (C) indicates the number of substitutions at each amino acid within the weblogo. The blue bar chart indicates RSV A while red dotplot indicates RSV B mutations. (For interpretation of the references to color in this figure legend, the reader is referred to the Web version of this article.)

The entropy of CCR (Sup. Table 1) between 163 and 189 amino acids accessed using BioEdit 7.2.5 version was 0.0039 (±0.005). The consensus sequence for hRSV A was *DFHFEVFNFVPCSICSNNPTCWAICKRIP* and *DYHFEVFNFVPCSICGNNQLCKSICKTIP* for hRSV B. WebLogos of reference sites for both RSV A & B indicate that only one site 178, experienced frequent mutations in RSV A (Fig. 1A) while RSV B (Fig. 1B) had multiple mutations at 176, 178, and 189 amino acid positions but in lesser frequency.

### 3.2 Phylogenetic analysis

**Fig. 2:**
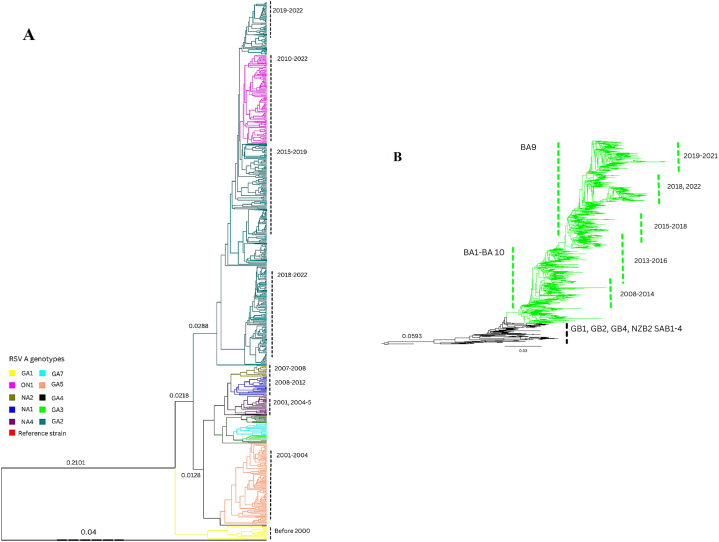
Phylogenetic tree of RSV G protein **Note:** Maximum likelihood trees of RSV A (A) & RSV B (B) describing the genotype divergence forming clades of new strains and their prevalence in respective years.

Maximum Likelihood tree for hRSV A (Fig. 2A), the GA1 genotype, which had a typical proline amino acid at site 215 along with substantial 101Pro, 111Ile, 121Gly, 123Lys, 141Ala, 157Ser, 258Leu, 262Met, and 293Pro which are considered as signature amino acid sites observed in the data. Leucine is found to be reverted to proline in GA2 strains typically found after year 2000. Further, the GA2 genotype shows 111Ser/Pro/Phe and 226His/Leu mutations while GA3 has 250Asn and 111Thr mutations. ON1 has an additional 72 nucleotide duplication, drifting the clade into a new sub-strain with higher spread feasibility. Changes at Pro286Leu, Leu274Pro, Pro293Ser, and Ser280Tyr splits the GA1 genotype branch to other GA genotypes (GA2-7). GA5 has additional substitutions Thr238Leu, Val225Ala, and Leu274Thr alterations that define the typical substitution diversity of the GA5 genotype. The average p-distance value calculated for RSV A is 0.032 (±0.001) while Tajima's D value was −2.054309(Sup. Table 1). The cluster picker shows similar clusters (Sup. Fig. 1).

Human RSV B (Fig. 2B) showed relatively lower but constant mutations that flowed from one genotype to the next at specific amino acid sites. ML tree was subdivided into two genotypes GB and BA. BA, prevalent since 2007, was further characterized into five subclades based on their collection year. These clades further split into distinct branches. Strains from 2018 to 2022 are clustered in one group, while strains from 2019 to 2021 are in a parallel group, and both are continuously evolving. From these clusters, the GB1 genotype included several distinctive mutations that include 137Ile, 150Ser, 152Ser, 208Ile, 222Met, 271Ser, and 280Ile, while GB2 has characteristic 223Thr mutation along with 118Thr, and 257Leu alterations. GB4 has two substitutions, namely Pro219Ser and Pro216Ser, as idiosyncratic indicators of this genotype. The genotype also has Lys224Arg, Lys234Glu, Lys258Asp, Asn230Asp, and Gln248Arg mutations in the G gene that differentiate it from the GB3 genotype that has characteristic Arg136Ile alteration. GB3 also exhibits single change substitution at 219 position that is proline to leucine instead of serine in the case of GB4. GB5 exhibited different mutations from time to time, referring to positive selection of specific amino acid changes. However, characteristically Leu237Ser/Pro and Thr143Asn were typical, while certain sub-strains isolated later on showed Ser291Lue and Glu305Asp that were due to missense mutation at 293 instead of a stop codon. SAB1 showed a change from serine to phenylalanine at site 277, along with 20 AA duplication sites located at 267, 269, and 270, present in SAB2 and SAB3, splitting them into sub-strains of GB3. Ala116Thr, Thr126Lys, and Gln262Leu/Pro were responsible for GB6 lineage, while GB7 has distinctive Gln180Arg, Arg214Ile, Arg242Lys, Val251Ile/Met, and Glu272Asp substitutions that were found in the analyzed dataset. Further, average p-distance value calculated for RSV B is 0.028 (±0.001) and Tajima's D value was −2.312741. Cluster picker shows similar clusters (Sup. Fig. 2).

### 3.3 Geographical distribution

**Fig. 3:**
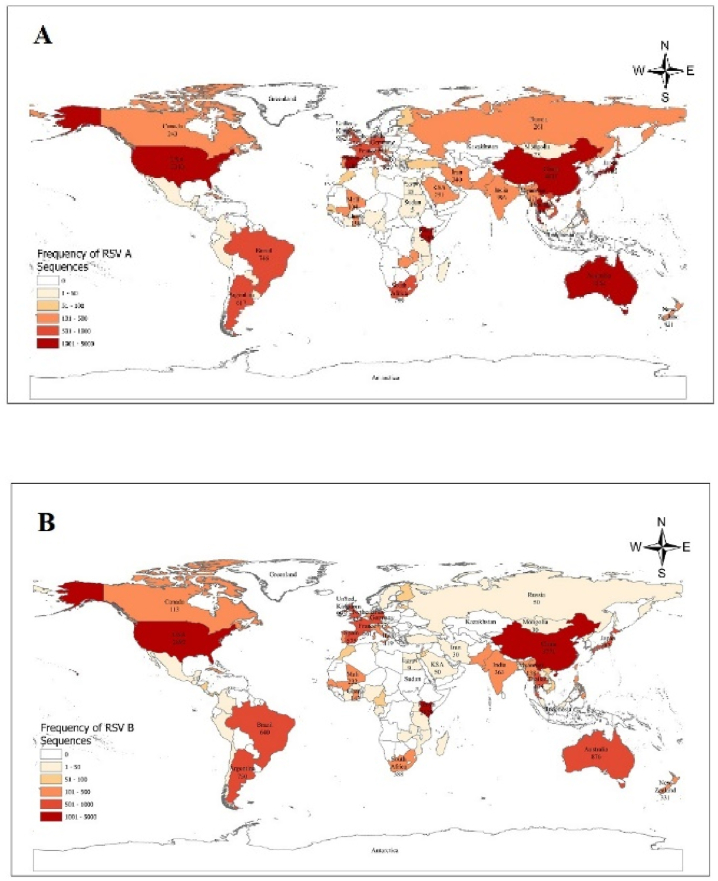
Geographical distribution of RSV G protein **Note:** Geographical representation of the frequency of hRSV A (A) and B (B) sequences reported from different geographical locations with the highest contributors, such as China and the USA.

The study infers that most of the sequences retrieved from NCBI and GISAID were recorded from China, followed by the United States of America, and then by Kenya for both RSV A and B. For instance, sequences retrieved from China were 4035(14.74 % out of 27363) and 3771(16.90 % of 22307), and from the USA they were 3310(12.09 % out of 27363) and 2892(12.96 % of 22307) for RSV A and B respectively. The frequency of distribution of all the sequences is presented in Fig. 3 (A & B).

### 3.4 Mutational analysis

**Fig. 4:**
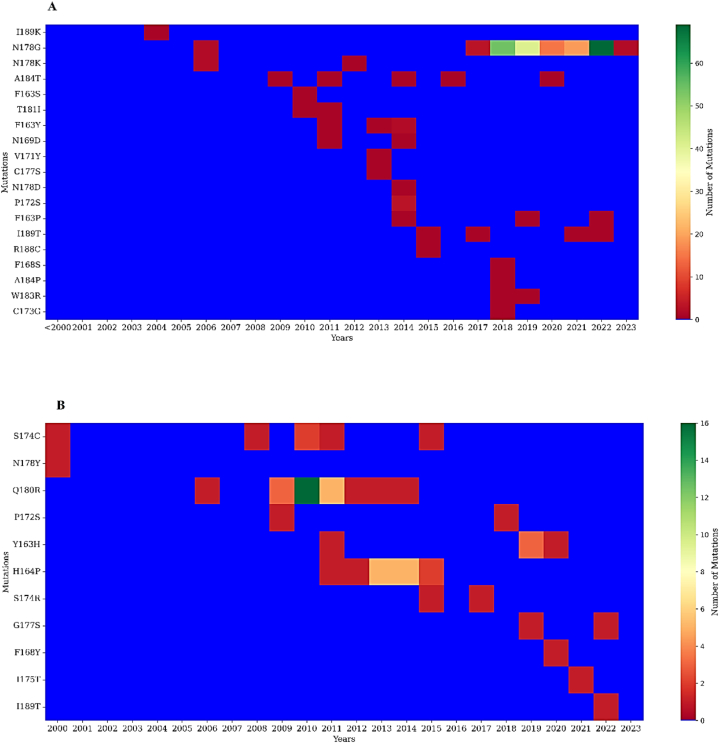
Heat maps of CCR region in RSV A & B **Note:** These heat maps represent mutations recorded year-wise in the CCR between 163 and 189 amino acids for hRSV A (A) and hRSV B (B).

For mutational analysis in CCR, we examined amino acids between 163 and 189 positions regarding G gene CDS from M74568.1 and AY353550.1 strains of hRSV A and hRSV B, respectively. In hRSV A (Fig. 4A), no mutations were observed until 2004, whereas alteration appeared at 189 position, changing lysine to isoleucine, followed by a couple of mutations N178K and N178G in 2006. Then a series of mutations appeared frequently, but the average entropy mentioned earlier showed that the number of mutations were negligible compared to the hypervariable region. The most frequent mutations were recorded at 178 positions, and the highest number was observed in 2022. For hRSV B (Fig. 4B), the 178 positions remained quite stable after recording several mutations until 2000 but sustained the conserved nature throughout. The highest number of mutations were observed at 180 position, changing from arginine to glutamine, which remained prevalent from 2009 to 2014 but later reverted. Another sustaining mutation for the 2011 to 2015 period was Pro164His, however, the number of mutations was limited.

Complete mutational analysis (291 amino acids) of G gene CDS (Sup. Fig. 3) revealed that the glycoprotein is highly prone to mutations and does not exhibit a sustainable tendency of AA proportions. The mucin-like regions have extensive mutations, while the CCR region shows a conserved state in both hRSV A & B strains. The initial 100 amino acids showed a non-homogeneous mutational trend, while the mucin-like domain had a relatively homogeneous flow of mutations.

### 3.5 Bayesian skyline analysis

**Fig. 5:**
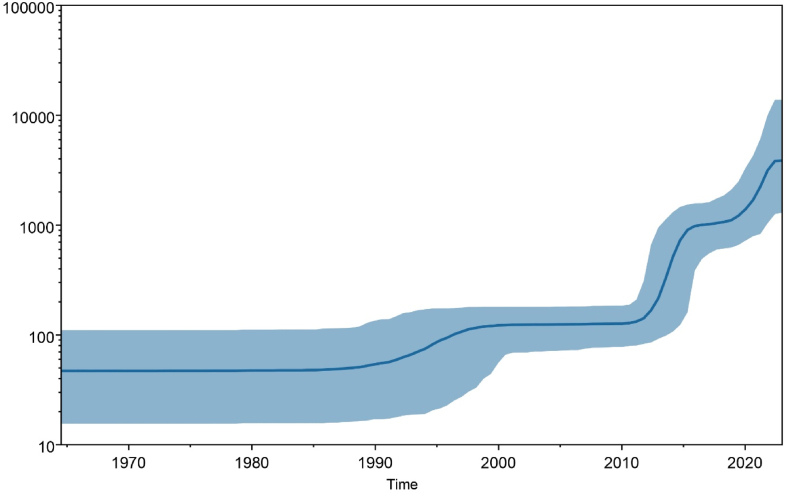

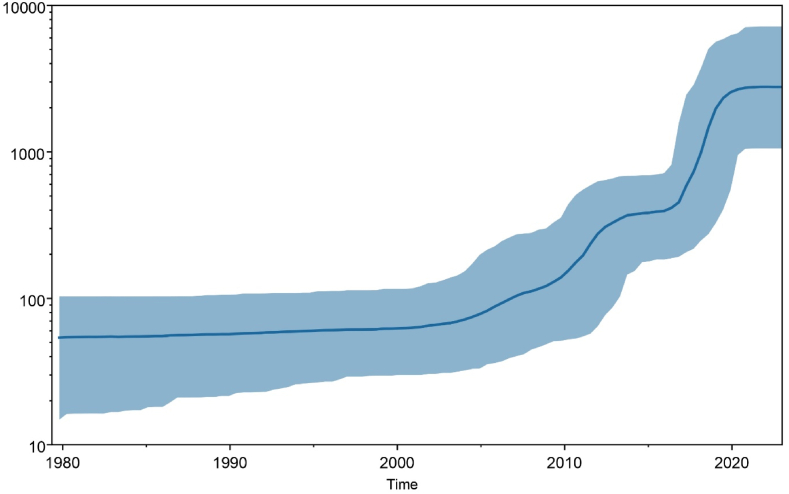
A Bayesian skyline plot of RSV A G protein. **Note:** BSP analysis indicating the population dynamics of RSV A G gene till 2022 with a solid blue line marking mean effective population with shaded blue region representing 95 % HPD. ?B Bayesian skyline plot of RSV B G protein. **Note:** BSP analysis indicating the population dynamics of RSV B G gene till 2022 with a solid blue line marking mean effective population with shaded blue region representing 95 % HPD. (For interpretation of the references to color in this figure legend, the reader is referred to the Web version of this article.)

The best-fit model used to estimate RSV A & B G gene adequate population size was the generalized time-reversible model (GTR) with a random relaxed molecular clock having a lognormal relaxed distribution. Bayesian skyline plot (BSP) of RSV A(Fig. 5A) with 95 % Highest Posterior Density (HPD) indicated that the population size of the G gene was relatively constant till 1990. Later, the viral genome started evolving, and adequate population size expanded rapidly between 2010 and 2015. A similar trend was observed in the BSP adequate population size of RSV B (Fig. 5B), where the population size started stretching around 2005 and is expanding rapidly to date. Higher expansion in adequate population size was observed between 2015 and 2020.

## 4 Discussion

This study provides deep insights into multiple significant aspects of the respiratory syncytial virus, including the phylogenetic analysis, understanding the conservancy of central conserved region, geographical distribution, and the mutational analysis of complete G glycoprotein. The geographical distribution of RSV strains indicates that USA and China hold the highest number of sequence records. A study to examine global transmission of RSV conducted by T.A. Shishir et al. indicates that USA, UK and Australia served as central nodes of transmission. These countries received higher number of visitors and cross-border travels indicating possible flush of strains to other parts of world. On the other hand, China has the highest population, which directly influences the number of cases recorded. Further, Russia acquired RSV A & B from Australia and UK but no outsourcing was observed due to lack of surveillance [18]. Moreover, Canada recorded ON1 strain of RSV in 2010 yet overall number of records indicates low surveillance studies [19]. Bayesian skyline plots of RSV indicates the population dynamics and changes in effective population sizes of each strain. RSV A population dynamics indicate an increase in effective population size of virus around 2010 to 2014, which contributed to evolution of ON1 substrain in 2010 [19], and its increased prevalence appeared in Europe during 2012–14 [20]. GA2 strain remained predominant in last two decades with higher evolution from 2017 to 2022 [21]. The BSP of RSV B showed a steadier evolutionary pattern until the wave erupted for RSV B BA-like strain in 2000 [22]. Strains including GB1, GB2, GB4 and NZB2, SAB1-4 genotypes remained prevalent in 1990s–2000s. Events of evolution occurred in BA sub strain of RSV B leading to BA9-like strains that emerged and spread during last two decades. BA1-BA14 like sub strains remained prevalent until 2015 after which BA9 substrain dominated throughout the world. Similar indications are supported by ML tree of RSV B (Fig. 2B) and studies conducted by J. Song et al. and J.-M. Yu et al. [5,23].

G glycoprotein is extensively variable, but a highly conserved region between 163 and 189 amino acid regions lies cysteine-rich residues in all strains of RSV, including hRSV [24] and bovine RSV (bRSV) [25]. This central conserved region (CCR) is flanked by mucin-like extensively variable regions (Sup. Fig. 4). The un-glycosylated CCR is a strictly conserved site with 13 amino acids having dual disulfide topology of cysteine cross linking 1–4, 2–3, making a partially overlapping noose, CX3C motif [26]. The four cysteines making the noose motif have strict conservancy and showed no mutation throughout the years. Mutational analysis of both strains showed that the region between 163 and 189 amino acids has an extremely low entropy of 0.0039 (±0.005) in this study, providing concrete evidence of the conserved nature of CCR along with inferring reference studies [26,27]. The rigidity of the structure in both bovine and human RSV glycoprotein showed a similar configuration using NMR [28]. This CCR contains a CX3C motif [3] that tends to interact with CX3CR1, an immune cell recruiter G protein-coupled receptor expressed by epithelial cells, neurons, dendritic, and smooth cells [29,30]. GAGS and heparan sulfate interactions are mediated by the G protein heparin domain in viral attachments in immortalized cells. However, heparan sulfate is not detectable in RSV infection facilitated with human epithelial cells, CX3CR1 and RSV G. The interaction between both, CX3CR1 and CX3C mediates disease progression [31,32]. Along with that, this extensive conservation property of CCR also provides a target for humanized monoclonal Abs that effectively neutralizes hRSV in epithelial cells of the human airway, forming relatively small neutralizing epitopes [30]. CX3C motif also has the property to alter the immune response by ameliorating the Abs along with a reduction in mucin production in vivo experiments [33]. CB017.5 and CB002.5 Abs are distinct epitopes with overlapping symmetry, harvested from RSV-recovered adults demonstrated that these Abs bind with CCR of G protein using peptide mapping based on alanine scanning [26]. However, the interactive model remains poorly understood. Certain studies suggested that CCR cysteine noose binds on the same structural bases as CX3CR1 binds with fractalkine, which is the natural ligand of epithelial CX3CR1 [34]. However, a structural mimic of CX3C motif of glycoprotein has a different homology as compared with fractalkine CX3C motif [35] suggesting that both have substantially different binding mechanisms.

The phylogenetic analysis revealed the formation of different clades in response to various mutations in the G gene of RSV. The phylogeny trees were analyzed based on the clade formation and mutations responsible for the evolution of new clades by the passage of time. There were notable yet typical mutations that were associated with the progression of certain RSV strains present in each RSV sub-strain. Certain bona fide sites, including replacement substitutions and putative positive selection sites, define lineages and genotypes within a sub-strain. Many other established studies concluded that certain signature mutations at different positions were responsible for drifting a genotype into a new clade/genotype, either due to positive selection of environmental re-enforcement or certain favorable benefits of a mutation. RSV A & B showed a similar characteristic mutational trend that follows the adaptation of the environment and continuously evolving till today [5,36,37]. Overall mutational analysis showed that G glycoprotein is highly versatile and hypervariable. Very few regions show slight sustainability, but most of the gene is well nifty. These drifts include missense mutations, the addition of nucleotides at different positions that result in the formation of different genotypes, mutation at 293 amino acid changing stop codon to other variable amino acids that resulted in the extension of G gene to 312 amino acids, and specific other mutations. These mutations and duplicate regions include the addition of 72 nucleotides in the ON1 strain of RSV A [38]and 60 nucleotides region in RSV B, BA strain in G protein at variable positions designated to their fitness advantage in widespread worldwide [39].

## 5 Conclusion

In conclusion, this study found that the most sequences available at GISAID and NCBI for hRSV belong to China, USA, Kenya, and certain European countries, suggesting a need for continued surveillance throughout the world, especially in underdeveloped countries. The phylogenetic analysis of ML trees showed the evolution of hRSV strains by the passage of time and specific signature mutations that drifted the genotypes into new sub-stains. The mutational analysis provided evidence of the hypervariable nature of the G gene and the CCR motif that is highly conserved. This flanked region can offer bases to drug targets along with the conserved fusion protein region that could provide higher stability and efficacy of target drug against hRSV, covering all strains in a unified manner. Demographic-based surveillance is required to understand the flow of genetic variations in the population to derive effective strategies for controlling viral spread.

## 6. Limitations

This study used published datasets that might contain biased information. The geographical distribution of sequences recorded on NCBI and GISAID have a biased nature as we observed during the survey that many of the sequences were reported to both databases, making duplicates and sometimes the quality of sequences was also compromised. Other studies also observed this by urging these data-sharing organizations to be open source and have strict publishing policies. From the observed data, China has the highest frequency of recorded sequences followed by USA and Kenya, and the trend was similar for both RSV A and B, which was relevant to a study conducted by AC. Langedijk [40].

## 7 Data availability statement

Data used in the study is available at NCBI Genbank and GISAID. Further, renamed and deduplicated datasets and their metadata will be provided upon request.

## Funding

This research was funded by the Open Foundation of National Virus Resource Center, grant number NVRC-PY-01, and the General project of Shanghai Jiading District Health Commission, grant number 2021-KY-21, and the Technical Support Talent Project from the Chinese Academy of Sciences.

## CRediT authorship contribution statement

**Muhammad Nabeel Amjad:** Validation, Software, Methodology, Formal analysis, Data curation, Conceptualization. **Jing Wang:** Writing – original draft, Validation, Formal analysis, Data curation. **Muhammad Awais Ashraf:** Writing – review & editing, Resources, Methodology. **Bei Shen:** Writing – review & editing, Visualization, Project administration, Formal analysis. **Ghayyas ud Din:** Writing – review & editing, Resources. **Muhammad Asif Raza:** Writing – review & editing, Resources, Formal analysis. **Muhammad Shoaib:** Writing – review & editing, Resources. **Lihuan Yue:** Writing – review & editing, Resources. **Lingdie Chen:** Writing – review & editing, Resources. **Huiting Xu:** Writing – review & editing, Funding acquisition. **Wei Dong:** Writing – review & editing, Funding acquisition. **Yihong Hu:** Writing – review & editing, Supervision, Resources, Project administration, Investigation, Conceptualization.

## Declaration of competing interest

The authors declare that they have no known competing financial interests or personal relationships that could have appeared to influence the work reported in this paper.
