# Supplementary material for: Evolutionary trends of respiratory syncytial viruses: Insights from large-scale surveillance and molecular dynamics of G glycoprotein
Source: Heliyon. 2024 May 9;10(10):e30886. doi: 10.1016/j.heliyon.2024.e30886 (PMC11112325; doi:10.1016/j.heliyon.2024.e30886)
Supplement: Multimedia component 3 [file mmc3.docx]

**Supporting Images**


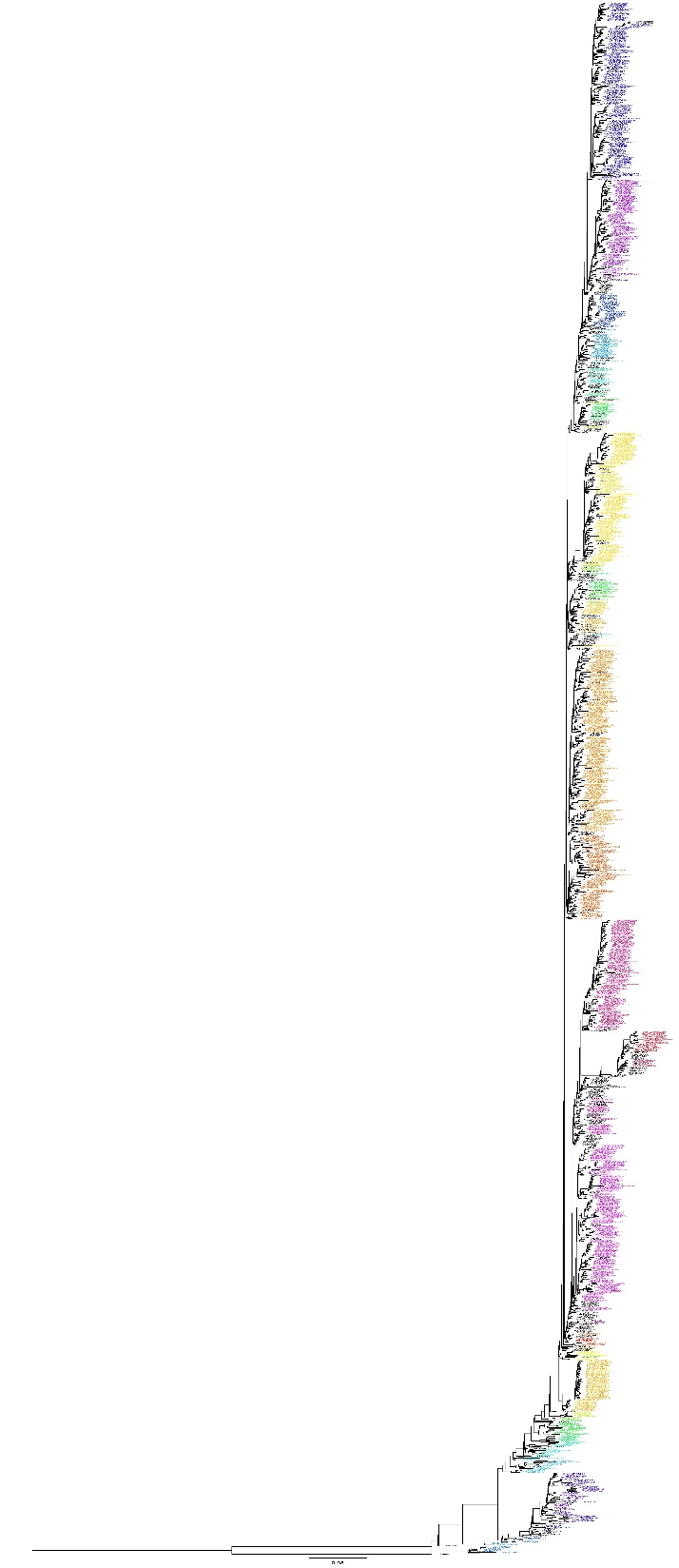


**Sup.** **figure: 1** ClusterPicker’s ML tree with colored clades for RSV A


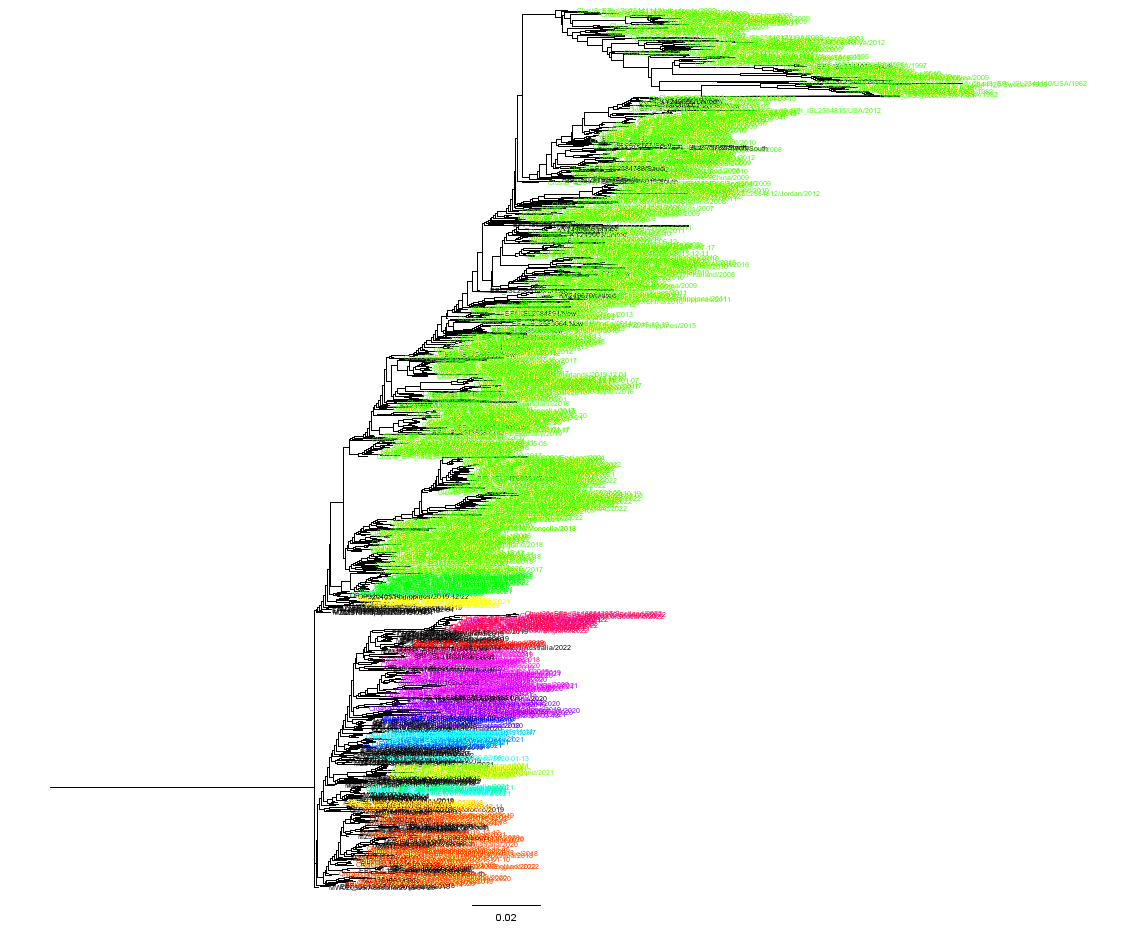
 **Sup.** **figure: 2** ClusterPicker’s ML tree with colored clades for RSV B


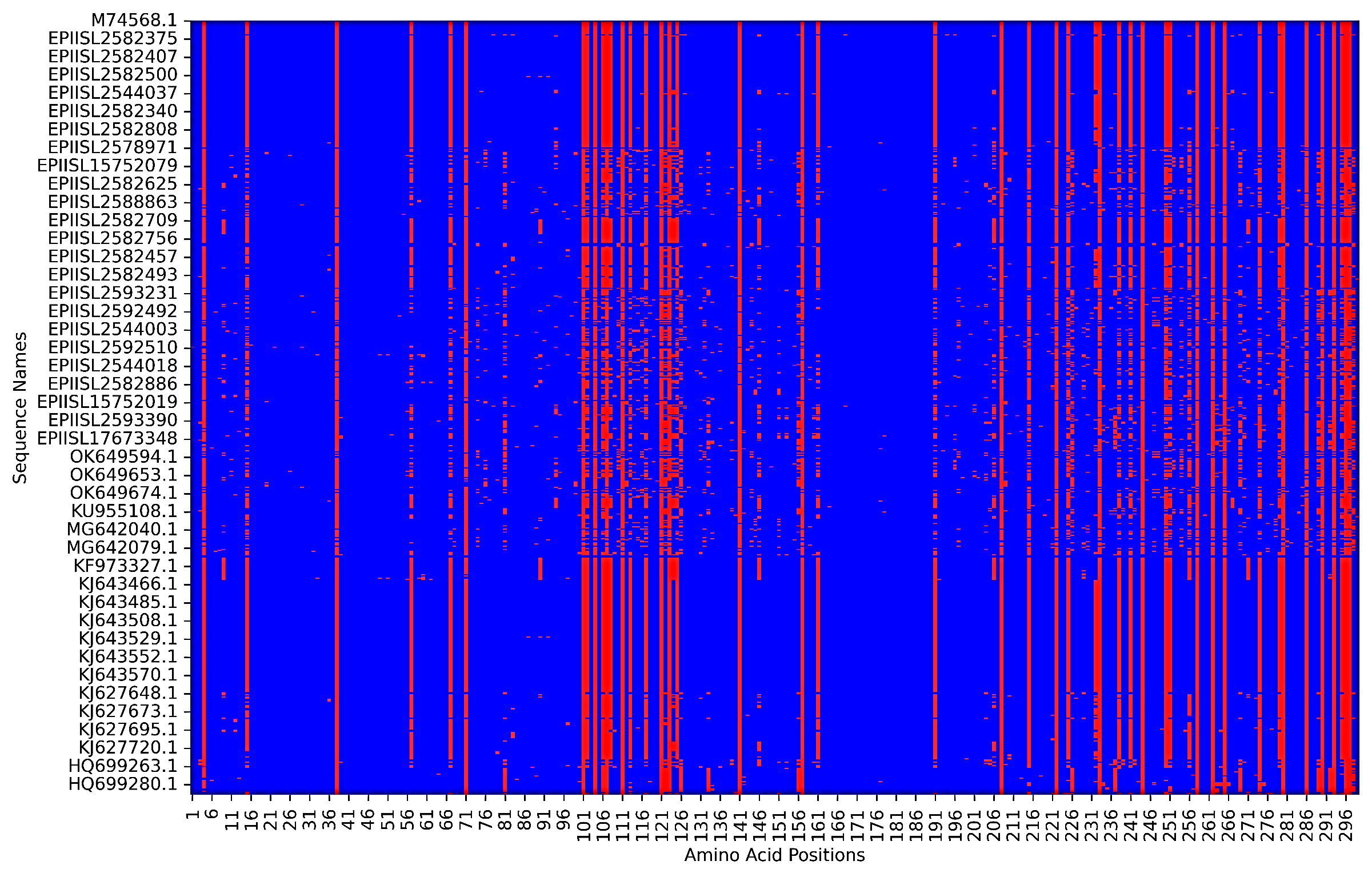

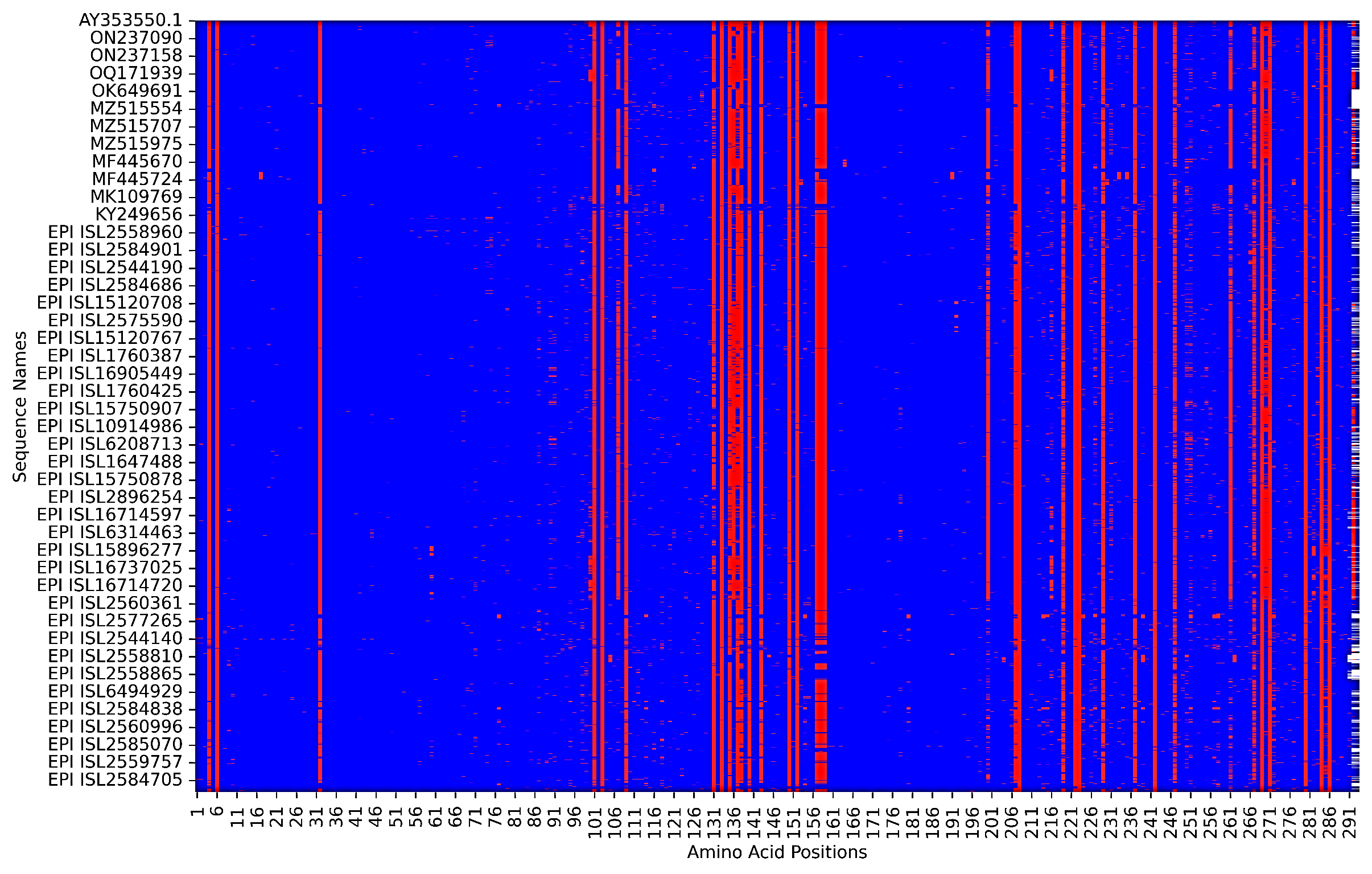


**Sup. figure 3.** These heat maps represent mutations recorded in a complete dataset of G glycoprotein after removing nucleotide duplications from different sites of complete CDS of hRSV A(left) and hRSV B(right).


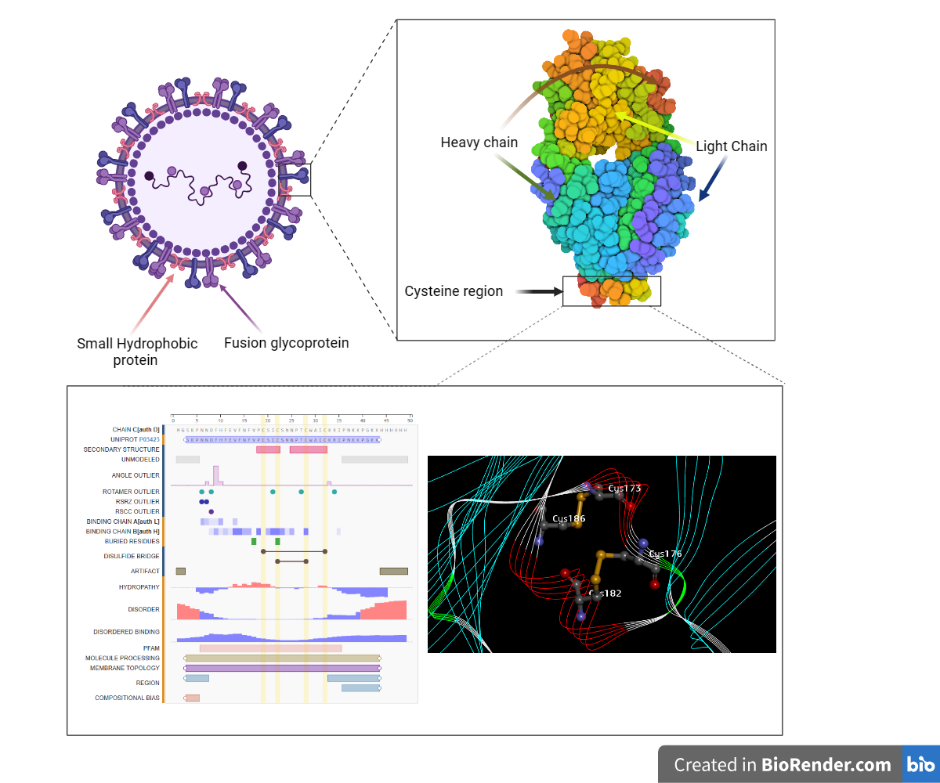


**Sup. figure 4**. The disulfide bonding bridges present between 173-186 and 176-182 cysteine amino acids with modelling data extracted from protein data bank [1].

Sup. Table. 1 Results from Tajima's Neutrality Test

| Strain | m | S | *p*_s_ | Θ | π | D |
| --- | --- | --- | --- | --- | --- | --- |
| RSV A | 2498 | 828 | 0.857143 | 0.102038 | 0.027716 | -2.054309 |
| RSV B | 2079 | 1119 | 0.297923 | 0.036259 | 0.006795 | -2.312741 |

*Abbreviations*: *m* = number of sequences, *n* = total number of sites, *S* = Number of segregating sites, *p*_s_ = *S*/*n*, *Θ* = *p*_s_/a_1_, *π* = nucleotide diversity, and *D* is the Tajima test statistic.

1. Fedechkin, S. O.; George, N. L.; Nuñez Castrejon, A. M.; Dillen, J. R.; Kauvar, L. M.; DuBois, R. M., Conformational flexibility in respiratory syncytial virus G neutralizing epitopes. *Journal of virology* **2020,** 94, (6), 10.1128/jvi. 01879-19.
